# THE-DB: a threading model database for comparative protein structure analysis of the *E*. *coli* K12 and human proteomes

**DOI:** 10.1093/database/bay090

**Published:** 2018-09-18

**Authors:** Justin S Diamond, Yang Zhang

**Affiliations:** 1 Department of Computational Medicine and Bioinformatics, University of Michigan, Washtenaw Avenue, Ann Arbor, MI, USA; 2 Department of Bioinformatics, Boston University, Cummington Mall, Boston, MA, USA

## Abstract

New methodology must be developed to improve the ability to characterize the growing number of amino acid sequences, which vastly exceeds the number of experimentally determined protein structures. Homologous proteins can be used as structural templates for modeling proteins that do not have experimentally determined structures. However, in many cases, there are no homologous proteins (typically <30% sequence identity) with determined structures from which a query sequence can be reliably modeled. The aim of protein threading is to use features, such as secondary structure, solvent accessibility and torsional angles, in addition to sequence patterns to identify structural templates from the protein databank to assist for full-length atomic-level structural modeling. However, there are still numerous protein sequences for which correct templates cannot be recognized. This raises the question as to what attributes allow query sequences to be matched to the correct but distantly homologous templates. To aid the investigation into this question and to provide genome-score protein structure for the biological community, a database called THE-DB (threading hard and easy protein database) has been developed in which it becomes possible to analyze over 15 000 query sequences from the Escherichia coli (*E*. *coli*) K12 and human proteomes, as well as to find their three-dimensional templates derived from the state-of-the-art threading algorithms which is not feasible with existing protein template databases. The *E*. *coli* K12 and human data can be downloaded in bulk from the THE-DB page.

## Introduction

Accurate modeling of protein structure and function has been one of the primary goals in modern computational biology research. The central dogma of biological function is the transmission of information from DNA to protein sequences via messenger RNA. Each protein sequence folds in a unique manner determined by physicochemical properties of the constituent amino acids of the sequence. The shapes of the protein folds, much like puzzle pieces, primarily determine interactions and binding affinities between different protein molecules. Hence, accurate prediction of protein structure helps in recognizing active sites of the molecules that in turn helps in developing drugs that can inhibit and activate certain molecules in a biological pathway.

There are currently three types of protein structure prediction algorithms that include *ab initio* folding (1, 2), threading (3, 6, 7, 8) and comparative modeling (4). Among them, the *ab initio*, or physics based, modeling is time consuming and not widely applicable as other due to decreased accuracy (5). On the other hand, comparative modeling (also called homology modeling), which attempts to model a query sequence based on homologous templates, can accurately predict structure of proteins that have templates with at least 30% sequence identity. However, homology modeling of proteins is often unreliable without homologous templates (<30% sequence identity). To fill this gap, threading algorithms are developed that can model proteins by identifying templates for a query protein sequence based on structural features such as secondary structure, solvent accessibility of the constituent amino acids and sequence profile information from multiple sequence alignment (MSA). (3, 6, 7, 8). Studies by Zhang and Skolnick have suggested the completeness of the protein databank (PDB) library (18, 19), even still, for certain query sequences, good templates cannot be accurately scored. So the threading problem becomes why are good structural templates not identified if they do exist? THE-DB (threading hard and easy protein database) was created to begin to address problem, which cannot be addressed with existing resources.

Some existing protein modeling databases currently available include the SWISS-MODEL (SM) (16) and the Protein Model Portal (PMP) (17). The primary purpose and use of these databases differs dramatically from THE-DB. The existing databases are formatted to find suitable templates for a query sequence of interest with comparative modeling. The results are a series of templates that are ranked by features such as sequence identity and quality of template. In other words, the purpose of these existing databases is to find accurate homologous templates for a protein sequence. In contrast, THE-DB was developed as a research tool with the primary function to progress the field of protein template identification. THE-DB is formatted for comparative analysis between different threading algorithms, which can be filtered by features such as template length, quality and more. This filtering mechanism is one of the features that are impossible in other databases. The functionality of THE-DB is suitable for visualization of protein templates, which can extrapolate on how threading algorithms identify good templates. With this, the goal of THE-DB is to aid in the development of new tools to understand the underlying complexities between the sequence to structure relationship that births the threading problem. Another significant difference is that existing databases rely almost exclusively on comparative algorithms (homology based) to identify templates. Algorithms like MUlti-Source ThreadER (MUSTER) (6) and SPARKS-X (8) were developed to identify non-homologous relationships between sequence and structure. Thus, THE-DB can also be used as a modeling tool if a query sequence shares no homologous relationships with other experimentally determined protein structures. In these cases, THE-DB will likely contain higher quality templates compared to SM and PMP. Put simply, THE-DB is a research tool to analyze the complexities of the threading problem by examining the results of state-of-the-art threading algorithms on the proteins of the human and Escherichia coli (*E*. *coli*) genome, while existing databases can be used as a look up table to find homologous templates for query proteins.

In order to recognize templates with correct folds for proteins, significant efforts have been put into the development of state-of-the-art threading algorithms. However, even the cumulative work of many threading (11) algorithms often cannot identify good templates for all proteins. Thus, a new approach must be developed to identify the characteristic(s) that remains hidden and keeps accurate template recognition at a distance. With the hope to identify the hidden characteristics and advance the state-of-the-art threading algorithms, THE-DB is created, where more than 900 000 individual threading templates, obtained from MUSTER (6), HHSearch (7) and SPARKS-X (8) threading programs, are deposited (Roughly 20 templates from each threading program per query sequence). In particular, this database enables quick and user-friendly lookup for the templates for the *E*. *coli* and human genomes while it would be time-consuming and cumbersome to do so on an individual level. These templates can be useful for protein modeling or comparative analysis, where the scientific community can distinguish characteristics between ‘hard’ proteins for which no good template can be found, and ‘easy’ proteins that have good templates.

To understand the complex relationships between sequence and structure, large-scale analyses should be taken to search for hidden patterns that remain elusive in protein threading. To understand this problem, it is important to remember that the sequential patterns of amino acid sequences contain all of the information necessary for a protein to achieve its native (lowest energy) conformation (16). This implies the threading problem is trivial; just pick the highest-scoring query and template pair given their alignment. This triviality fails when there exist no homologous relationships. In these cases, the best template for a given query sequence is often not the highest scoring. Thus, the current metrics used to characterize protein sequences are incomplete. The question to be asked is then, why is our current metric incomplete? Answers to these types of questions will inevitably bring about better identification methodologies, where a systematical analysis of the large-scale modeling such as THE-DB will be essential.

## Methodology

THE-DB uses a nonredundant PDB library with a cutoff of 70% sequence identity (https://zhanglab.ccmb.med.umich.edu/library/) to search templates for the proteins. Different threading algorithms use different features for template detection. The implications of these features are briefly described here as pertaining to the three algorithms, MUSTER, SPARKS-X and HHSearch, which are chosen as representative threading algorithms in this study. First, MUSTER, developed by Wu and Zhang (6), uses a scoring function with six terms, including sequence profiles from PSI-BLAST (13) derived MSA, secondary structure by PSI-PRED (14) (for query) and STRIDE (for template), structural profile, solvent accessibility and backbone dihedral torsional angles, to match the query and template. While SPARKS-X also uses profile–profile comparison and secondary structure, solvent accessibility and torsional angle match between query and template sequences, it does not use structural profile information (8). Finally, HHSearch uses sequence profile and secondary structure information to create a hidden Markov model (HMM) of the query which is compared against an HMM database of template structures (7). These three algorithms are chosen due to their relatively high performance compared to others, as well as because of their distinct scoring functions to identify structural templates from query sequences.

The template score is characterized by *Z*-Score:

(1) \begin{equation*} Z- Score=\frac{R^{\prime } score-\lt{R}^{\prime } score\gt}{\sqrt{\lt{R^{\prime}}^2 score\gt-\lt{R}^{\prime } score{\gt}^2}} \end{equation*}

Here, *R’* is the maximum of *R/L_partial_* or *R/L_full_*, where *L_full_* includes ending gaps in the query sequence and *L_partial_* does not. *R* is the raw alignment score, which is optimized against false positives in the LOMETS (20) paper to identify a cutoff score for each threading program. The *Z*-Score correlates how significant a template matches the actual structure of the query sequence. Thus, the higher the *Z*-Score the more likely the template accurately resembles the query structure. The *Z*-score cutoff values for THE-DB are 6, 7 and 11 for MUSTER, SPARKS-X and HHSearch, respectively. It is important to note that individual *Z*-Scores between different programs cannot be directly compared because of the distinct scoring functions of the programs. In-depth discussion of these scoring functions and cutoff parameters can be found in the corresponding studies. Based on the threading results by the programs, each query sequence can be categorized into one of three groups: easy, medium or hard. A query is identified as easy if all the three algorithms identify good templates for the query based on *Z*-score, while it is considered as hard if none of the programs detect any good template. If one or two of the algorithms find a good template for the query, it is regarded as medium.

The *Z*-score is an important metric in determining if the highest-scoring templates are easy or hard. Ideally, to determine if a template is good, the TM-score (16) would be used to calculate the topological similarities between query and template structures given an alignment between them. However, threading is used when the query structure is not determined experimentally, so the TM-score cannot be used because there is no reference structure for the query sequence. For this reason, the *Z*-score was developed to predict the quality of a query-template pair as it is correlated with the TM-score (6). In cases where the top templates share very little structural similarities, the *Z*-score is likely to determine if the template is good.

**Figure 1:**
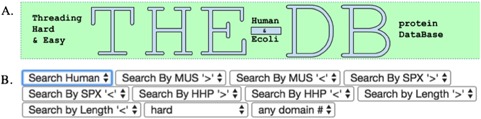
The title of the database (A) and the search filters for queries (B) are shown.

**Figure 2:**
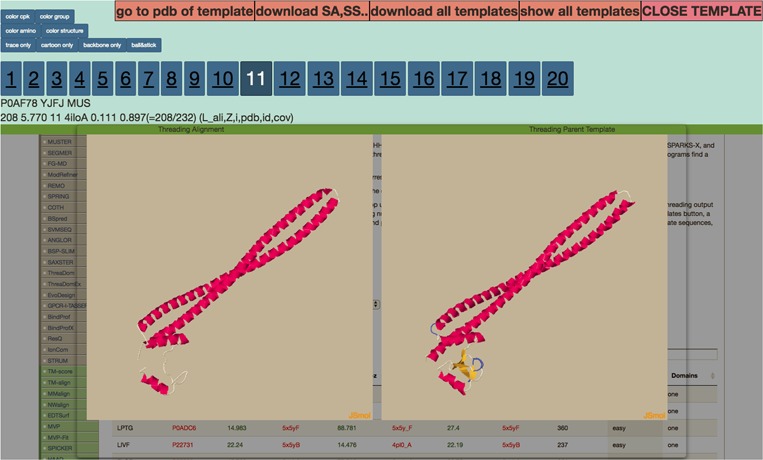
Display after the user clicks on the *Z*-score column for any threading algorithm. It visualizes threading output to the left and parent template on the right. The numbers 1–20 are clickable that route the users to the corresponding ranked threading templates.

**Figure 3:**
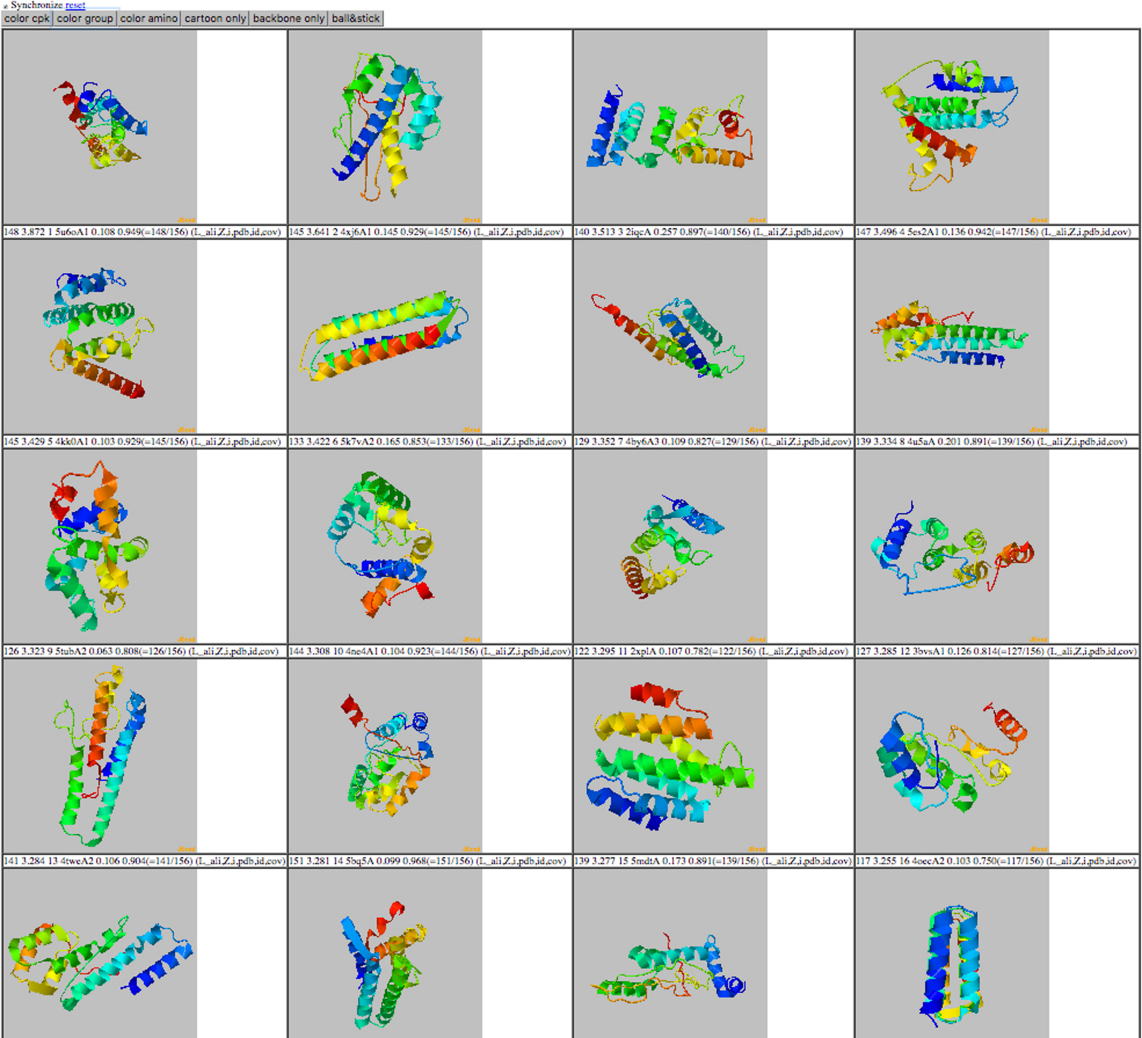
By clicking on the ‘show templates’ button in the modal view, a new page is loaded which displays all 20 threading templates at once. By pushing the sync button at the top of the page, the rotations of all the templates are in sync. Display all 20 threading templates at once after clicking the ‘show templates’ button in the modal view. Selection of the ‘synchronize’ button at the top of the page allows the rotations of all the templates in sync.

The features of THE-DB help to make it a significant contribution to the field of threading. Figure 1 shows the title and search filters of THE-DB (11, 12). The columns for THE-DB are PDB and Universal Protein Resource (UniProt) ID of the *E*. *coli* and human genome proteins, PDB IDs and corresponding Z-Scores of the first ranked templates identified by the three algorithms: protein length, threading type and number of domains of the proteins. Users can be directed to the UniProt profiles of the proteins by clicking their UniProt names. Similarly, by clicking the template names, users can access the corresponding PDB profiles. A modal page appears to visualize the threading templates (Figure 2) by clicking the *Z*-score. It is also possible to view the top 20 templates (Figure 3) obtained by each threading algorithm on a single page by clicking the ‘show all templates’ button. This feature allows the user to look at the structural features that were significant in the identification of these highest-scoring templates, and to see the progression of how the scores change with different structural features. The key features that differentiate THE-DB from other protein model databases is it has filters that rank all of the items in the database by a specific column feature (*Z*-score, length, etc…), it allows for visualization of the top templates on the same page, and it explicitly shows the alignment between the query and template sequences. All of which are not possible with the use of the SM or the Protein Data Portal. THE-DB also has a browsing option, which allows the user to find subgroups of query-template pairs without identifying a specific query sequence of interest. This can be useful when the user wants to analyze certain traits of query-template pairs without searching by single sequences. Other options include downloading the PDB structure files for the threading outputs and parent PDB templates.

## Results

About 4280 proteins from the *E*. *coli* K12 proteome and over 12 514 proteins from the human proteome were collected to construct THE-DB. These proteomes were selected because the human proteome is of great interest for medical care, and the *E*. *coli* proteome is well categorized in literature and has a large impact in understanding microbial interactions. These selected proteins include almost all UniProt *E*. *coli* sequences (www.uniprot.org/proteomes/UP000000625), and a representative fraction of UniProt homo sapien sequences (www.uniprot.org/uniprot/?query=proteome%3Aup000005640), including subcellular proteins less than 1000 amino acids long, with evidence of protein existence. A representative selection of human proteins is filtered with UniProt’s search function based on the following criteria: expression levels detected and subcellular proteins. Although two-domain proteins have slightly more complex folds for which finding good templates is typically difficult, we still include two-domain proteins in THE-DB for completeness, since the purpose of this database is not to display accurate structural templates, but to find ways to improve current state-of-the-art threading algorithms. However, very few two-domain proteins are identified as hard targets, since both domains must be hard to label the proteins hard.

The backend of THE-DB is done with MYSQL databases to look up data, and PHP script to send the data to the users. There are two databases, one for human proteins and another for *E*. *coli*. The data is filterable by *E*. *coli*/human proteomes, *Z*-score of all three threading algorithms, length of the query proteins, domain count and type (easy, medium or hard) of the proteins. When the *Z*-score from a threading algorithm for a query protein is clicked on, the visualization of the threading output can be seen on the left with the original template PDB structure on the right for that particular threading algorithm. The output of each program is the top 20 ranked templates, which are displayed by clicking on the numbers. The images are displayed using JSmol software (5, 9). Below the image, more data corresponding to the query-template alignment as well as the predicted secondary structure of the query sequence are shown. Solvent accessibility, pair constraints and fasta alignment information can also be viewed by clicking on the radio button above the search filter.

During the creation of THE-DB, 499 out of 4280 *E*. *coli* proteins are identified as hard targets, while 881 and 3040 proteins are regarded as medium and easy targets, respectively. On the other hand, out of 12 514 human proteins, 918 and 2938 are identified as hard and medium targets, respectively, while the rest of them are identified as easy targets. Figure 6 shows the relative proportions of easy, medium and hard targets in *E*. *coli* and human. The *E*. *coli* proteome contains 64.7% easy and 12% hard, while the human data set contains 64.1% easy and 10.5% hard targets.

The data indicate that ∼10% of the human and *E*. *coli* proteomes cannot be accurately threaded. While Skolnick and Zhang have shown that there is at least one good template for each query protein (18, 19), it raises the question what characteristics play roles to hide these templates from being detected as good templates.

**Figure 4:**
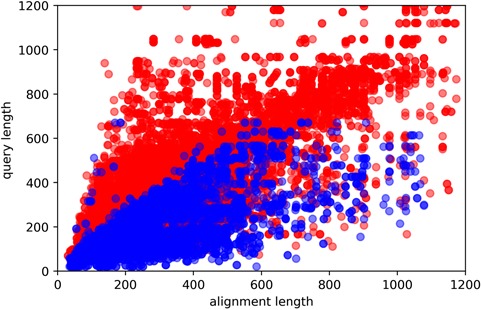
Here, *y*-axis is the query sequence length, while the *x*-axis being the template sequence length. Red and blue colors indicate easy and hard targets, respectively. Plotted are all of the query sequences with best 60 template matches from all algorithms.

To distinguish further THE-DB from PMP and SM; specific examples will be shown in which the non-homologous template identification algorithms that THE-DB is dependent on, MUSTER and SPARKS-X, are predicted to contain better quality templates based on *Z*-score for THE-DB, the reliability measure for PMP and the all atom measure of the model quality estimate for the SM. Although these measures are not directly comparable, they will help distinguish the functional purpose of THE-DB from PMP and SM which can be simply stated as THE-DB is based on non-homologous relationships between query sequence and template due to MUSTER and SPARKS-X, whereas the latter two are based on homologous relationships which is dependent upon high sequence identity for good results. Thus, the cases in which THE-DB houses better templates compared to other existing databases will likely be those cases in which no high sequence identity template exists for a query of interest. The following examples were found by searching THE-DB for sequences where the *Z*-score of SPARKS-X and MUSTER were higher than their corresponding *Z*-score threshold, but HHSearch was not. The reason for excluding HHSearch is that it does not highlight the distinct differences of THE-DB because it is a comparative algorithm, which is commonly used for homologous relationship detection in the existing databases. In contrast, MUSTER and SPARKS-X results are exclusively found in THE-DB.

The sequence with the UniProt identification ID of P15029, P23876, P37095, Q7Z4T9, Q9BT92^*^ and Q96M91^*^ is just a small fraction of cases that satisfy the above criteria (^*^containing quality templates in THE-DB and contains no existing template in PMP and/or SM). As well, by checking in PMP and SM, their models were shown to be of medium or poor reliability. These results suggest that THE-DB is in a unique position to contain better quality templates in cases of low sequence identity. As THE-DB is representative of the majority of the *E*. *coli* proteome, it can be used to much success when the best quality template is desired. The human dataset of THE-DB contains only a representative portion of the proteome, which makes it slightly less applicable for finding good templates of human proteins.

**Figure 5:**
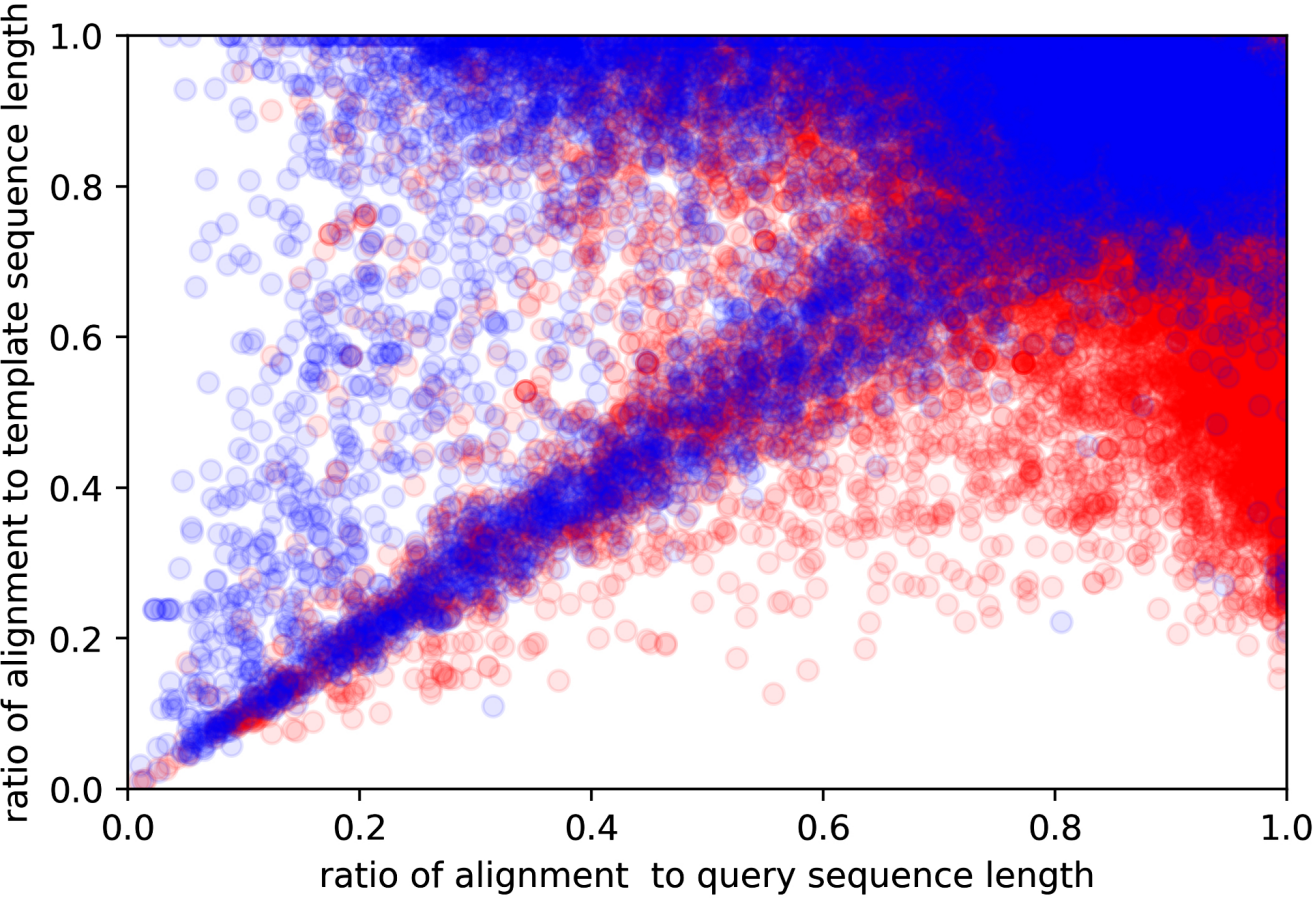
Here, *y*-axis is the coverage for the template sequence (alignment length/template sequence length), while the *x*-axis being the coverage for query sequence (alignment length/query sequence length). Red and blue colors indicate easy and hard targets, respectively. Plotted are all of the query sequences with best 60 template matches from all algorithms.

**Figure 6:**
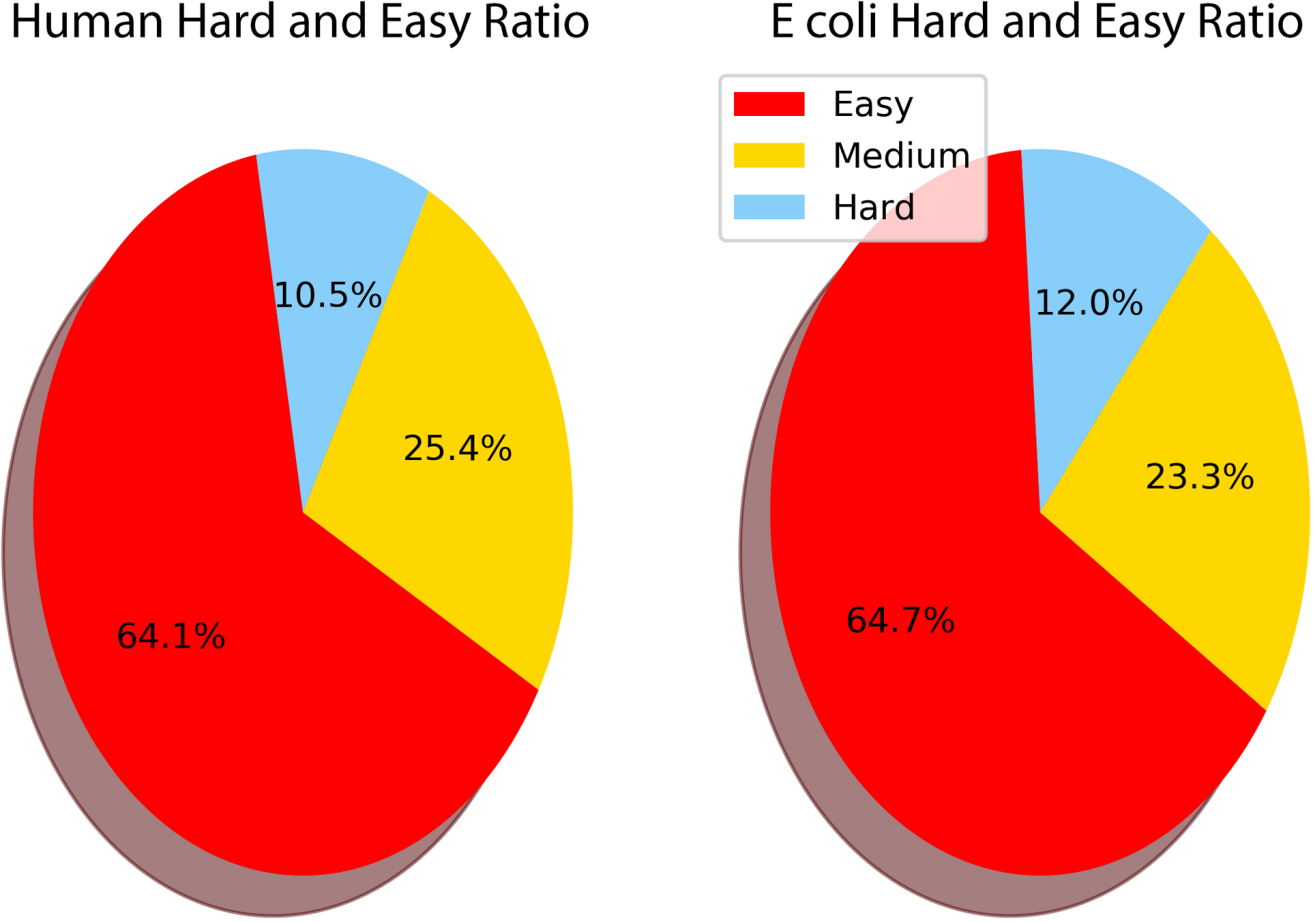
Ratio of easy, medium and hard targets in the human and *E*. *coli* proteomes. Two-domain query sequences are not included as they are characterized as easy if one of the domains is identified as easy. The sequences of length smaller than 30 are also not included as they have a higher chance of aligning to a template by chance.

**Figure 7:**
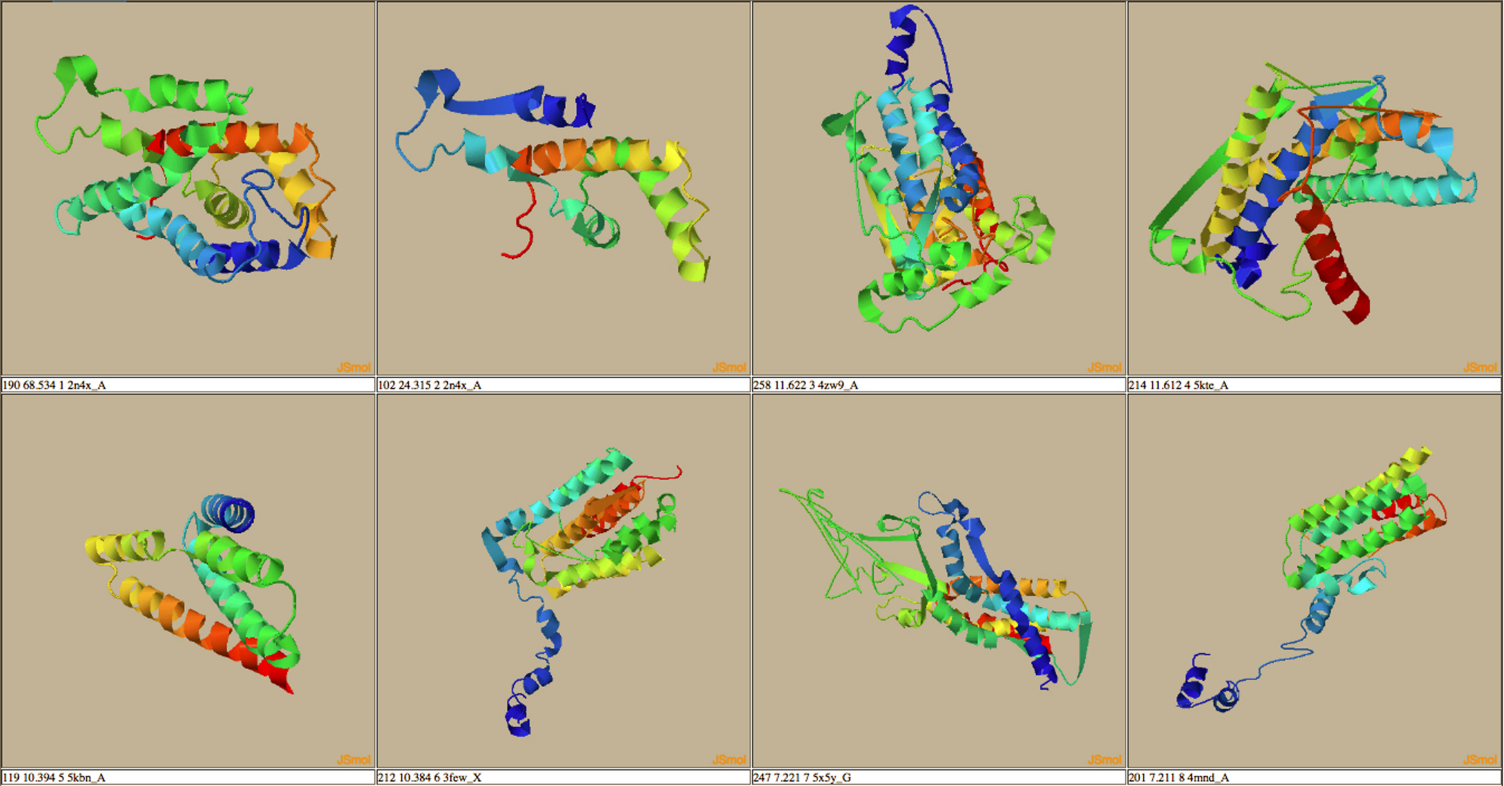
Top eight threading alignments from HHSearch for the protein RCNA P76425.

## Discussion

THE-DB is a tool to help overcome the current plateau in fold recognition algorithms. It allows users to easily visualize and analyze easy, medium and hard targets from the human and *E*. *coli* K12 proteomes. Three questions must be asked to improve fold recognition methodologies. First, what trends are exhibited between hard and easy targets? Second, what is the reason for different trends? Third, how can these trends be implemented to improve threading? Protein threading algorithms can be improved by finding better reasoning for these questions.

Approximately 10% of the human and *E*. *coli* proteomes are categorized as hard targets. In particular, hard targets are frequently matched to templates with a bigger ratio of template sequence length to the query sequence length, while easy templates tend to have the inverse relationship (Figure 4). A possible explanation is that smaller template fragments create a better reference for query structure modeling. This could also explain why shorter sequences are more commonly hard targets as smaller sequence length means it is more likely to match to templates of larger sequence sizes. The size differences between template and query sequence length could essentially mean that even though they may share somewhat similar sequences, their overall structural topologies do not match. Our study also shows that coverage alignment could be another key characteristic, where higher coverage to template sequence length is more prevalent in hard targets (Figure 5).

**Figure 8:**
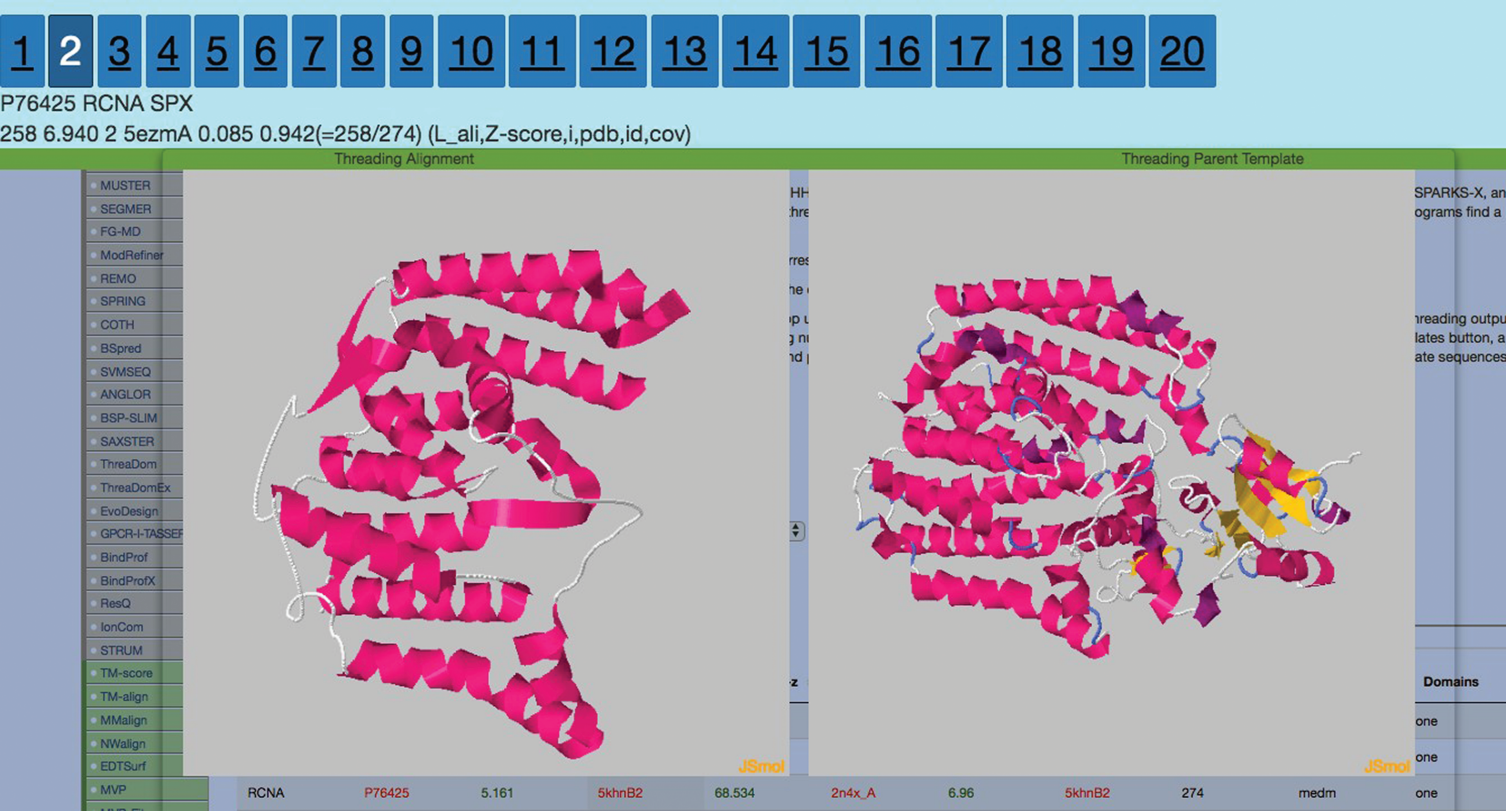
Second best threading template for RCNA P76425 from SPARKS-X.

A possible route to analyze the data in THE-DB is to pick a subgroup of proteins and analyze the structures based on recurrent patterns. One possible subgroup of interest would be proteins of length between 100 and 300 that are labeled as hard. This group of proteins can bring insights into why so many of proteins around these lengths are categorized as hard. To complement those proteins, the proteins with the same length but categorized as easy or medium could be analyzed. For instance, RCNA P76425 with a length of 274 is labeled as medium since HHSearch identifies a good template, while MUSTER and SPARKS-X fail to hit any good templates for this protein. Figure 7 shows the first two template structures of the protein that are classified as good with a score much higher than the cutoff for HHSearch. The general trend from these structures shows that the lower the score the more the structures open up, and the alpha helices become longer. Overall, the actual protein mainly consists of alpha helices. In Figure 8, the second best template for SPARKS-X is shown. The *Z*-score is just below the threshold, which could be caused by the beta-sheet regions that manipulate the structure differently compared to structures with only alpha helices. One hypothesis is that some of the best-scoring templates for hard targets are incorrect because of sections of the template structure that are not aligned to the query sequence causing the template to maintain a distinct fold.

The previous examples can lead to new hypotheses like the following; although regions outside of the threading alignment could cause a protein to fold differently, this is not reflected well in scoring functions. Based on observations from THE-DB, a possible research topic would be the development of an additional ‘negative’ scoring function that considers how ‘poorly’ a sequence matches to a structural template. This could complement the conventional scoring function by filtering out false positive templates.

Overall, the development of the THE-DB database has provided a starting point for careful data analysis and an ease of addressing difficult questions regarding threading. Although THE-DB was primarily developed as a tool to guide further research into threading, it can also be a useful database for the biological community who needs structural information of the proteins in *E*. *coli* and human genomes.

## Notes

**Database URL:** https://zhanglab.ccmb.med.umich.edu/THE-DB/
